# The Dynamic Optical Breast Imaging in the Preoperative Workflow of Women with Suspicious or Malignant Breast Lesions: Development of a New Comprehensive Score

**DOI:** 10.5402/2012/631917

**Published:** 2012-09-27

**Authors:** Massimiliano D'Aiuto, Giuseppe Frasci, Maria Luisa Barretta, Adolfo Gallipoli, Giovanni Maria Ciuffo, Flavia Musco, Sergio Orefice, Viviana Frattini, Ilves Guidi, Claudio Siani, Emanuela Esposito, Anna Crispo, Maurizio Montella, Andrea Chirico, Giuseppe D'Aiuto, Aldo Vecchione

**Affiliations:** ^1^Department of Breast Disease, National Cancer Institute “G. Pascale” Foundation, Via Mariano Semmola, 80136 Naples, Italy; ^2^Department of Radiology, National Cancer Institute “G. Pascale” Foundation, Via Mariano Semmola, 80136 Naples, Italy; ^3^Breast Unit, Clinical Institute Zucchi, Via Zucchi 24, 20052 Monza, Italy; ^4^Breast Unit, Clinical Institute Pio X, Via Francesco Nava 31, 20159 Milano, Italy; ^5^Breast Surgery Divison, Medical Institute Monterosa, Via Monterosa 3, 20149 Milano, Italy; ^6^Breast Prevention Area, Physios Clinic, Via Chiesa Nord 52, 41016 Modena, Italy; ^7^Epidemiology Division, National Cancer Institute “G. Pascale” Foundation, Via Mariano Semmola, 80136 Naples, Italy; ^8^National Cancer Institute “G. Pascale” Foundation, Via Mariano Semmola, 80136 Naples, Italy

## Abstract

*Purpose*. To determine the diagnostic accuracy of DOBIComfortScan in patients with Breast Imaging Reporting suspect breast lesions (BI-RADS) 4-5 breast lesions. *Materials and Methods*. One-hundred and thirteen patients underwent DOBIComfortScan examination before surgery. Twelve parameters were taken into consideration to define DOBI findings. *Results*. Twenty-seven radical mastectomies, 47 quadrantectomies and 39 wide excisions, were performed. Overall, 65 invasive cancer, 9 in situ carcinoma and 39 nonmalignant lesions, were observed. Ten out of 12 considered parameters resulted significantly in association with histology at discriminant analysis. A summation score of 30.5 resulted to be the best cut off at ROC analysis, giving a sensitivity and specificity of 80% and 87%, respectively, and a positive predictive value of 92.2%. Finally the following DOBI-BI-RADS model was developed: malignant B5 ≥ 38 score); possibly malignant (B4 = 25 − 37 score); benign but the possibility of malignancy cannot be excluded (B3 = 20 − 24 score); benign (B2 < 20 score). *Conclusion*. definition of other parameters permits to improve the accuracy of this procedure. Further studies are warranted to define the potential role of DOBIComfortScan in breast cancer imaging.

## 1. Introduction

Breast cancer is the most frequent female malignancy in developed countries. In Italy almost 40,000 new cases were diagnosed in 2010, with more than 7,000 deaths [1]. There is a general agreement about the correlation between tumor size and survival; therefore, a delay in diagnosis may negatively affect the prognosis [2]. Mammography screening has played an important role in the reduction of breast cancer mortality that has been observed in the past two decades, although in a recent report it has been stated that the improvements in treatment and in the efficiency of healthcare systems might be plausible explanations for that [3]. In any case, mammography cannot be considered an ideal tool to achieve early diagnosis. Indeed, the density of the breast in younger women can represent a major obstacle for the detection of small tumors; moreover, mammography has limited indication in women with breast implants [4, 5]. Breast MRI has become an essential diagnostic procedure in high-risk women, but it cannot be considered an ideal tool for large scale screenings [6]. 

The use of transillumination of the breast dates back to the 1920s, when it was proposed to investigate breast cancer [7]. However, the low sensitivity and specificity of transillumination limited its clinical use. With progress in photonic technologies and mathematic modeling of light propagation through tissues, the optical imaging has evolved and new technologies are now available [8].

The dynamic optical breast Imaging (DOBI) has recently shown to be effective in detecting breast cancer in young women [9]. The DOBI with ComfortScan technology is based on detecting and analyzing red light transmission through the breast tissue and recording the transitory responses of the tissue due to a compression that induces changes in blood-flow volume. This pressure stimulus results in the dynamic behavior of optical properties of the tissue creating various dynamic profiles in regions with abnormal vascularization compared to the vascularization of normal breast tissue. The ComfortScan system discriminates between breast regions with abnormal vascularization, considered suspect for malignancy, and those with normal tissue [10].

In a recent study the DOBI ComfortScan was evaluated in 46 patients with Breast Imaging Reporting and Data System (BI-RADS) 3–5 breast lesions. The DOBI ComfortScan examination showed statistical difference in number of suspect pixels between benign (*n* = 12) and malignant (*n* = 35) lesions (1325 versus 3170, resp., *P* = 0.002). In this population optical imaging had a sensitivity of 74%, specificity of 92%, and diagnostic accuracy of 79% [11]. 

The present multicenter study aimed at increasing the diagnostic accuracy of DOBI ComfortScan in a larger population and at assessing whether it can be a useful tool for breast cancer screening.

## 2. Materials and Methods

### 2.1. Patient Characteristics

We retrospectively reviewed the medical records of patients who had undergone surgical excision of suspicious or positive breast lesions at the National Cancer Institute of Naples and at the Clinical Zucchi of Milan between March 2009 and July 2011. All patients had been considered eligible for surgery since they showed BI-RADS 4-5 breast lesions at standard imaging (mammography and breast ultrasound). Patient's workout included medical history, breast abdominal and pelvic ultrasound, mammography, chest X-ray, and routine blood chemistry with tumor markers assessment (CA 15.2 and CEA). Cytological or microhistological characterization of the lesions was also acquired. Breast magnetic resonance imaging (MRI) was performed in case of suspicious multicentric and/or bilateral breast cancer. Bone scan was also included in the staging in case of malignancy. All patients who underwent Dynamic Optical Breast Imaging in the preoperative workflow were considered eligible for the study. Four patients were excluded due to insufficient illumination of the breasts. The study was approved by the local ethics committees and was performed in accordance with the current version of the Declaration of Helsinki and the International Conference on Harmonization of Good Clinical Practice Guidelines. Informed consent was obtained from all patients after the nature of the examinations was explained fully.

### 2.2. The Dynamic Optical Breast Imaging

The DOBI Medical ComfortScan system is an advanced digital imaging device that uses high-intensity, light-emitting diodes (LEDs) and gentle external pressure to highlight areas with vascular abnormalities. The high-intensity LEDs transmit red light through the breast, if the light encounters a neoangiogenic region, it is absorbed or scattered differently than in other regions of the breast. This is caused by the different concentrations of oxygenated and deoxygenated hemoglobin. 

The system is made of three physical assemblies (Figure 5): the C-arm assembly, the controller, and the computer system. The details of the technique have been previously described [9–11].

### 2.3. Image Acquisition and Processing

The patients stand in front of the machine and the breast is positioned onto the panel of the C-arm assembly. The LEDs of the system illuminated the breast and the light (with wavelength of 640 nm) is transmitted through the tissues and quantified on the other side by the charge coupled device (CCD) camera. In the acquisition window of the operating software, the operator can mark the region of interest (ROI) placing two pointers indicating, respectively, the nipple and where it is supposed to be the lesion.

A soft transparent silicone membrane is placed in contact with the upper surface of the breast and then inflated under computer control during the exam. The pressure is set to 5 mmHg for the first 15 s of the scan, raised to 10 mmHg over the next 30 s (the dynamic image sequence), and decreased back to 5 mmHg for the final 15 s. Forty-five frames are overall acquired (five baseline before applying pressure and 40 during the dynamic sequence). The transmitted light is detected by the CCD throughout the scan and recorded by the computer that processed the data to generate the dynamic angiogenic signature (DAS), a sequence of images of the breast in a cranial-caudal view. 

The analysis of the Dynamic Angiogenic Signature may identify changes in local blood perfusion and oxygen saturation as variations in image contrast defined area of pathologic interest (API). The API presents an increase of both blood volume and depletion of blood oxygen with a reduction of the amount of light reaching the CCD camera. This feature appears like an area of decreased intensity (dark blue) or highly decreased intensity (purple) of color contrast. The ComfortView software selects the optimal contrast “auto contrast” for display of the study varying the scale (percentage) used along the *y*-axis; however, it is possible to zoom in on the focused area by changing the scale of the *y*-axis in dynamical mode [12].

### 2.4. Criteria of Exam Interpretation

**Figure 1:**
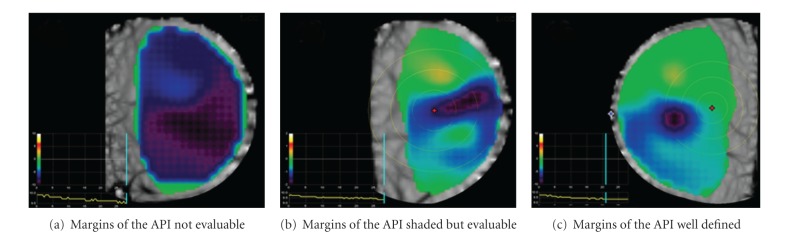
Morphologic features of the area of pathologic interest (API).

We defined twelve items to correctly estimate the API in order to standardize the criteria of the exam interpretation. The parameters were blindly evaluated and grouped into three categories: morphologic, spatial, and temporal items (Table 1). Morphologic items evaluate the major diameter and margins of the API. The absence of API means absence of detectable neangiogenesis as well as a very diffuse blue indicates an area not well evaluable (Figure 1(a)). If the blue area is more bowl-like (some transition, but with a large, even-colored area of deep blue instead of a smaller, more focused area at the epicenter), it is not as likely associated with malignancy and is not clearly benign, thus, it is considered indeterminate (Figure 1(b)). A focal, intense API with relative steep transitions of the margins (like an inverted mountain peak, or “peaked”) is more likely to be associated with malignancy (Figure 1(c)). 

The spatial items define the distance of the API from the nipple marker and its location in the ROI marker. The lesion is more relevant when enclosed in the ROI marker and localized distant from the nipple to avoid the “nipple blue-phenomenal” due to the natural convergence of the vessels to the nipple. The maximum pixel intensity of the API also correlates with malignancy. An intensity of −4000 is considered predictive of malignancy, while −2050 to −4000 is less strongly predictive, but still likely malignant. An intensity of 0 to −2050 is considered indeterminate (consistent with a “flat” or nearly flat curve). 

**Figure 2:**
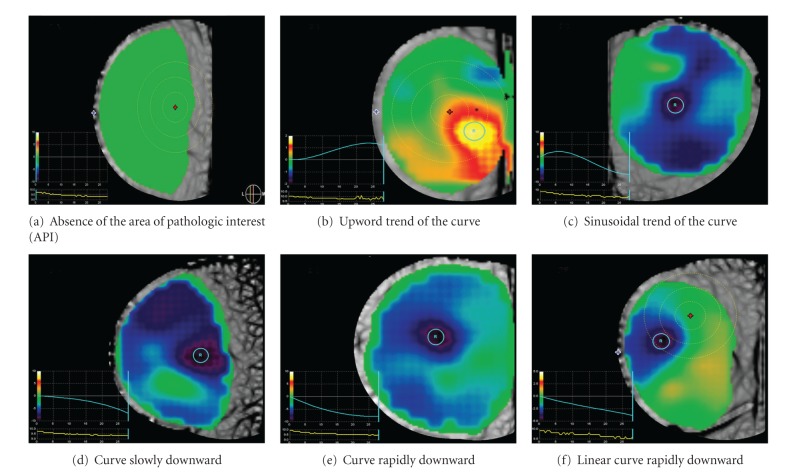
Temporal signature graft curves. The blue line indicates the temporal signature graft curves. The pressure graph is displayed under the temporal signature graph (Yellow line).

The temporal items evaluate the time of API detection and its duration and the temporal signature graft (in auto and zoom mode), which represents the changes over time of the pixel intensity as well as the differences of the temporal signature grafts between the API and the normal breast tissue in auto and zoom mode. The absence of API means absence of detectable neangiogenesis (Figure 2(a)).

Curve becoming more positive over time (trend is upward) indicates that less light is being absorbed and is consistent with a benign area. This would appear as an area of green that becomes orange/yellow/white over the sequence of images (Figure 2(b)). Curves that are very wavy (sinusoidal or highly variable in amplitude) are considered benign (Figure 2(c)).

**Figure 3:**
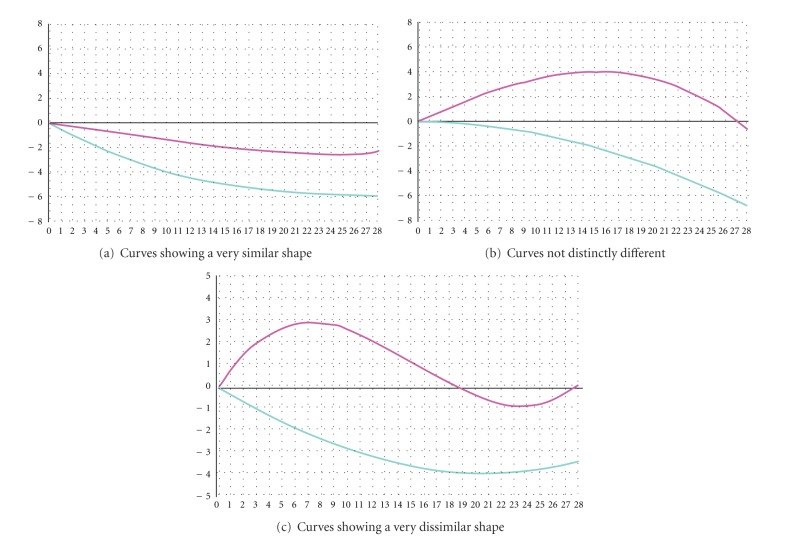
Comparison between temporal signatures of the API (blue curve) with a control marker placed on a non-pathologic area of the breast (red curve).

If the curve is becoming more negative over time (the trend is downward), this indicates that more light is being absorbed and is consistent with the presence of malignancy. A more rapid decrease of the curve is more likely associated with malignancy (Figures 2(d) and 2(e)). A linear downward trend represents the feature most predictive of malignancy (Figure 2(f)). Additional information can be drawn from the comparison of the temporal signatures of the API with the remaining breast tissue (control marker). If the API and the control marker curves are very similar (the background is “behaving” consistently and the lesion ROI has the same pattern), especially when the curves are trending upward, this is consistent with an area that is likely benign (Figure 3(a)). If the lesion ROI is in an area of malignancy, that area is expected to respond to pressure differently from the uninvolved breast. Very dissimilar lesion and, or ROI curves are more often associated with an area of malignancy (Figure 3(c)). A highly variable curve throughout the background (no distinct reference curve pattern) or a lesion ROI curve that is not distinctly different than the reference ROI curve is considered indeterminate (Figure 3(b)). Also, an area of malignancy is expected to become relatively more intense over time compared with the uninvolved area of the breast; therefore, diverging curves (a lesion ROI curve that diverges from the reference ROI curve) indicate an area that is likely malignant. Overall these findings may be evaluated in auto-selected mode as well as in zoom mode [13]. 

### 2.5. Statistical Analysis

We scored the 12 variables using a standardized code (Table 2) and then evaluated the relationship of these scores with histology through the discriminant analysis (DA).

DA was used to model the value of a dependent categorical variable based on its relationship to one or more predictors. We calculated the Wilks' lambda for each variable to define its correlation with histology (benign versus malignant). The variables that resulted significantly associated with histology at discriminant analysis were recoded with a new score according to the strength of their correlation. 

The summation score derived from the discriminant analysis was evaluated by using the receiver-operating characteristic (ROC) curves to assess the best cutoff. The area under ROC curve (AUC) illustrates the performance of a classifier over all sensitivity and specificity levels. The AUC can range from 0.5 (chance performance) to 1.0 (perfect performance). In order to minimize the rate of false negative, we decided to take into account a performance at very high sensitivity level.

Finally we developed a 4-class DOBI-level correlating with pathologic findings: malignant (category B5); possibly malignant (category B4); benign but the possibility of malignancy cannot be excluded (category B3); benign (category B2).

The chi-square test was used to assess the relationship between the DOBI score and prognostic variables.

All of the statistical analyses were performed using SPSS (version 16.0).

## 3. Results

### 3.1. Pathologic Findings

Overall 113 women underwent DOBI-ComfortScan before surgery. Median age was 49.8 (range 25–79) years. Twenty-seven radical mastectomies, 47 quadrantectomies, and 39 wide excisions were performed in the 113 patients reviewed. Overall, 65 invasive cancer, 9 in situ carcinoma and 39 nonmalignant lesions, were observed. Fifty-three patients out of 74 patients with carcinoma presented positive axillary nodes. Pathological tumor size was T1, T2, and T3 in 41, 20, and 4 patients, respectively. Histotype of patients with invasive carcinoma was as follows: ductal carcinoma 54, lobular carcinoma 6, others 5. At immunochemistry, 54 patients had HR positive, 16 patients presented an HER2 overexpressing, and 4 cases were triple negative tumor Table 2.

### 3.2. Optical Imaging

Discriminant analysis of the DOBI Scan results was validated originating a classification matrix with 80% of original grouped cases correctly classified and 85.0% of cross-validated grouped cases correctly classified, having confirmed a major contribution of the same variables that had significant correlation coefficients.

The relationship between the categories and score of various items is shown in Table 3. 

The following variables better discriminated the function: major diameter; pixel intensity; temporal signature graft in auto mode; temporal signature graft in zoom mode. 

**Figure 4:**
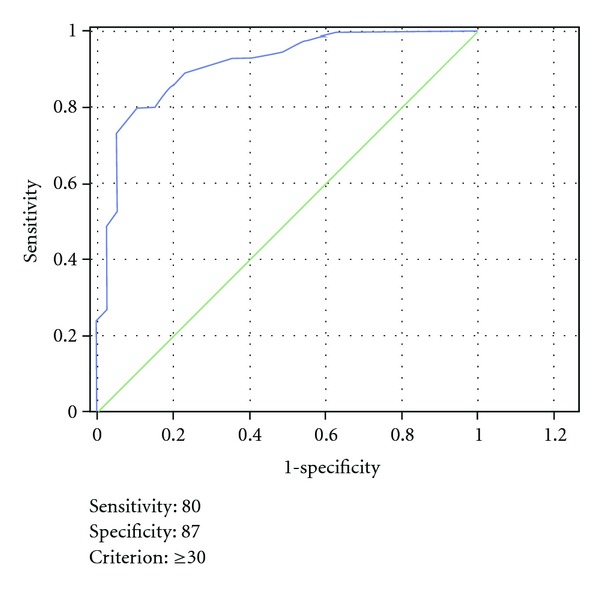
The Roc curve.

**Figure 5:**
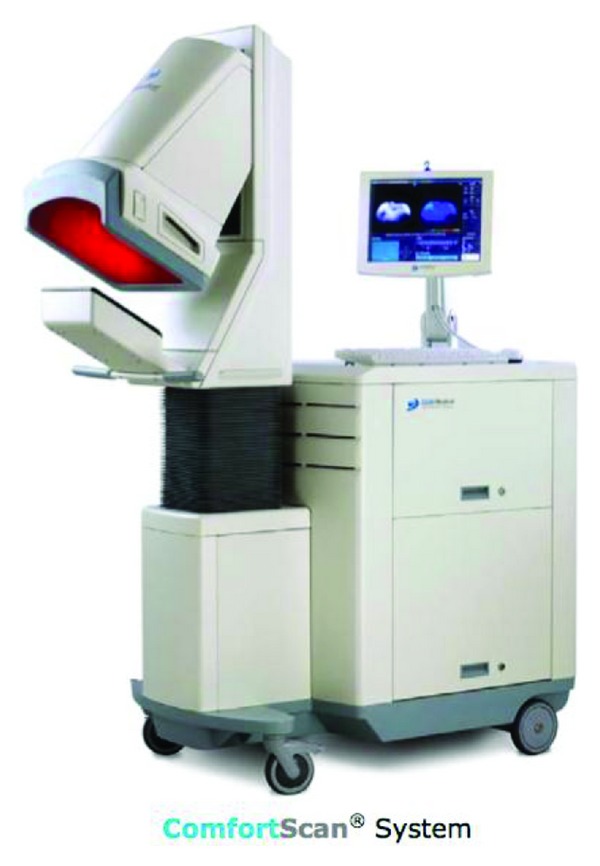
The DOBI ComfortScan System device.

The summation score, made up of 10 variables (two were excluded since they resulted not significant), was evaluated by using the receiver operating characteristic (ROC) curves to assess the best cutoff; these are plots of the true positive rate against the false positive rate for different possible cut-off points (Figure 4). The best cut-off DOBI score for tumor detection was 30.5, giving a sensitivity and specificity of 80% and 87%, respectively. Overall, 64 patients had a >30.5 DOBI-Score; 59/64 had a tumor, resulting in a positive predictive value of 92.2%. Fifteen out of 49 patients with DOBI score <30.5 had a malignant lesion, which resulted in a negative predictive value of 69.5%. In order to maximize the capability of DOBI in identifying benign lesions we considered a lower score. The DOBI-Score cut-off 25 better permitted the identification of benign lesions. Twenty-one out of 24 women with <25 score showed a benign lesion (negative predictive value 87.6%). Seventy-one out of 89 patients with score > 25 had cancer (positive predictive value 80%). 

Finally, the DOBI score was related to diagnostic results (DOBI Level) such as malignant (category B5 ≥ 38 score); possibly malignant (category B4 = 25–37 score); benign but the possibility of malignancy cannot be excluded (category B3 = 20–24 score); benign (category B2 < 20 score). 

Table 4 shows the diagnostic categories. The chi-square analysis showed no statistically significant correlation between the categorical DOBI score and T size (*P* = 0.7), grading (*P* = 0.5), Cerb2 (*P* = 0.3), and receptor status (*P* = 0.2) (Table 2). 

## 4. Discussion

Breast cancer is the first cause of cancer-related death among women in western countries. In 2010, the standardized mortality rate was estimated to be 13.3/100.000 worldwide and 30.9/100.000 in developed countries [14]. One-third of these cancer deaths can be avoided by early detection and appropriate therapy; this means that nearly 400.000 lives could be saved every year [15]. Current investigation of breast cancer is performed in women between 50 and 70 years of age by X-ray mammography, sometimes supplemented by ultrasound and/or magnetic resonance imaging (MRI) [16]. Mammography has a sensitivity generally ranging between 79% and 89%; however, its diagnostic accuracy is much lower in radiographically dense breasts that limits its usefulness in high-risk young women [17–19]. The dynamic optical breast imaging (DOBI) may identify breast tumors by detecting changes in local blood perfusion and oxygen saturation due to neoangiogenesis. The DOBI has numerous potential advantages, including the use of nonionizing low energy light radiation, high sensitivity, continuous data acquisition for real-time monitoring, and low cost. To date, very few papers investigating the potential use of the DOBI ComfortScan system for the early detection of breast cancer are available. Athanasiou et al. reported the results of DOBI scan in a series of 72 patients with BI-RADS 4-5 breast lesions [20]. The interpretation of the optical imaging was based on the analysis of the following parameters: the presence of early, focal, intense blue color in the area of interest, the pixel intensity of the blue areas, and the type of temporal signature of dynamic curves. A numeric level of suspicious score was calculated based on all these elements and a score > 5 was considered suspicious. At histological analysis, 49 out of 72 lesions were found to correspond to malignancies and the DOBI recognized 30 of them. Among them, 30 corresponded to malignant lesions and 11 to high-risk benign lesions. Eleven out of 19 false negative cases observed were ductal carcinoma in situ. However, the criteria of imaging interpretation were not clearly defined and there was a high interobserver variability in the determination of the score. Fournier et al. evaluated the DOBI system in 47 BI-RADS 3–5 breast lesions [11]. A statistically significant difference in numbers of suspect pixels between benign (12) and malignant (35) lesions was observed (*P* = 0.002). The ROC curve showed that the optimal cut-off value for the number of pixels was 2050. Twenty-six out of 35 malignant lesions (sensitivity = 74%) and 1/12 benign nodules (specificity 92%) showed a number of pixel > 2050. Interestingly, 6 out of 9 malignant lesions missed by DOBI scan had been classified BI-RADS 5 by mammography, therefore correctly described as most certainly malignant. In view of that, the authors stated that mammography and optical imaging should be considered complementary, as they describe different physiological properties of tissues. 

The purpose of our study was to improve the accuracy of DOBI ComfortScan by evaluating other parameters besides maximum pixel intensity. We considered 12 items, grouped in three categories, spatial, temporal, and localizing since we hypothesized that the temporal changes of the pixel intensity as well as the spatial characteristics of the API and its relationship with normal breast could add useful information to achieve a correct diagnosis. The discriminant analysis permitted to exclude two spatial items, the location of the API in the ROI marker and its distance from the nipple. The remaining ten items, which had significantly correlated with histology, were recoded according to strength of their correlation. A higher score was given to the following five parameters which better discriminated the function: major diameter, pixel intensity, and temporal signature graft in auto and zoom mode as well as the correlation between the temporal signature grafts of API and normal tissue. The ROC analysis showed that the best cutoff of DOBI score was 30.5, which resulted in a sensitivity and specificity of 80% and 87%, respectively. A DOBI score > 25 resulted in a much higher sensitivity (93%) and a DOBI score > 21 reached a 99% sensitivity although the specificity dramatically fell. According to the performance depicted in the ROC curve, we developed a DOBI 0–5 categorical classification (DOBI-level, Table 4) resembling the classical BI-RADS categorization. The DOBI-level 4-5 (score > 25) permitted to correctly identify 71/74 (96%) malignant lesions, with only 18 false positive findings. A DOBI-level 2-3 (score ≤ 25) correctly identified absence of tumor in 21/24 (87%) cases. Overall, a cutoff of DOBI score 25 resulted in a positive predictive value and negative predictive value of 80% and 87%, respectively. Both the positive and negative predictive values we observed in the present study compare favorably with those previously reported. Interestingly, the univariate analysis showed that age, T size, invasive/in situ, histotype, grading, HR status, Ki 67, and HER2 status did not significantly affect diagnostic accuracy. In view of that, DOBI ComfortScan could be a very useful diagnostic tool in young women. We must remark that this was a retrospective study, thus further confirmations in prospective trials are required before considering the DOBI ComfortScan a useful tool for early detection of breast cancer. In order to assess whether the DOBI could be used for breast cancer detection in asymptomatic women, a large prospective randomized trial comparing this methodic with other standard breast imaging examinations should be carried out. There are some limitations that must be taken into consideration. The DOBI ComfortScan allows the acquisition of breast images only in craniocaudal projection and this may avoid the detection of cancers localized in the axillar pilaster and/or mammary sulc. Moreover, DOBI ComfortScan could be not feasible in women with very small, firm breasts that could not be properly illuminated as well as in women previously submitted to surgery and/or bioptic procedures, due to the presence of residual edema and extravasation. The possible limiting role of factors like inflammatory breast conditions, skin breast tattoos, menstrual cycle phases, and vasculitis should be addressed in appropriate trials. 

## 5. Conclusion

The DOBI ComfortScan is a low-cost, noninvasive technique with a good potential for discriminating benign from malignant lesions. According to our results, the definition of other parameters besides pixel intensity permits to improve the accuracy of this diagnostic procedure. Further studies are warranted to define the potential role of DOBIComfortScan in breast cancer imaging.

## Figures and Tables

**Table 1:** Criteria for exam interpretation.

Class of item	Characteristic of API^1^	Description	Figure
Morphologic	Margins	Absence of API^1^	Figure 1(a)
		Shaded	Figure 1(b)
		Well defined	Figure 1(c)
	Margins in zoom mode	Absence of API or margins indistinct	
		Shared or well defined	
	Major diameter	Absence of API^1^ or API > 5 cm	
		From >3 to 5 cm	
		From 1 to 3 cm	

Spatial	Location of API^1^ in the ROI^2^ marker	Absence of API or out of the rings	
		Within the third ring	
		Within the second ring	
		Within the first ring	
	Distance of the API from the nipple marker	≤2 cm from the nipple	
		>2 cm from the nipple	
	Maximum pixel intensity	<2050	
		From 2050 to 4000	
		>4000	

Temporal	Time of API detection	Absence of API	
		From 10 to 20 seconds	
		<10 seconds	
	Duration of API at the dynamic analysis	Absence of API or <10 seconds duration	
		Duration from 10 to 20 seconds	
		Duration >20 seconds	
	Temporal signature graft in auto mode	Absence of API	Figure 2(a)
		Curve with upward trend	Figure 2(b)
		Sinusoidal curve	Figure 2(c)
		Curve slowly downward	Figure 2(d)
		Curve rapidly downward	Figure 2(e)
		Linear curve rapidly downward	Figure 2(f)
	Temporal signature graft in zoom mode	Absence of API	
		Curve with upward trend	
		Sinusoidal curve	
		Curve slowly downward	
		Curve rapidly downward	
		Linear curve rapidly downward	
	Similar/dissimilar temporal signature grafts in auto mode	Absence of API or curves very similar	Figure 3(a)
		Curves slightly different	Figure 3(b)
		Curves very dissimilar	Figure 3(c)
	Similar/dissimilar temporal signature grafts in zoom mode	Absence of API or curves very similar	
		Curves slightly different	
		Curves very dissimilar	

^1^API: area of pathologic interest; ^2^ROI: region of interest.

**Table 2:** The DOBI score derived from the discriminant analysis.

Class of item	Characteristic of API^1^	Code	Description	Figure
Morphologic	Margins	0	Absence of API^1^	Figure 1(a)
		1	Shaded	Figure 1(b)
		2	Well defined	Figure 1(c)
	Margins in zoom mode	0	Absence of API or margins indistinct	
		1	Shaded or well defined	
	Major diameter	0	Absence of API1 or API > 5 cm	
		1	From >3 to 5 cm	
		3	From 1 to 3 cm	

Spatial	Maximum pixel intensity	0	<2050	
		2	From 2050 to 4000	
		4	>4000	

Temporal	Time of API detection	0	Absence of API	
		1	From 10 to 20 seconds	
		2	<10 seconds	
	Duration of AIP at the dynamic analysis	0	Absence of API or <10 seconds duration	
		1	Duration from 10 to 20 seconds	
		2	Duration >20 seconds	
	Temporal signature graft in auto mode	0	Absence of API	Figure 2(a)
		1	Curve with upward trend	Figure 2(b)
		2	Sinusoidal curve	Figure 2(c)
		3	Curve slowly downward	Figure 2(d)
		6	Curve rapidly downward	Figure 2(e)
		9	Linear curve rapidly downward	Figure 2(f)
	Temporal signature graft in zoom mode	0	Absence of API	
		1	Curve with upward trend	
		2	Sinusoidal curve	
		3	Curve slowly downward	
		6	Curve rapidly downward	
		9	Linear curve rapidly downward	
	Similar/dissimilar temporal signature grafts in auto mode	0	Absence of API or curves very similar	Figure 3(a)
		3	Curves slightly different	Figure 3(b)
		6	Curves very dissimilar	Figure 3(c)
	Similar/dissimilar temporal signature grafts in zoom mode	0	Absence of API or curves very similar	
		1	Curves slightly different	
		2	Curves very dissimilar	

**Table 3:** Association between DOBI score and some prognostic factors.

	DOBI score				
Patient characteristics	<30,5		≥30,5		*P* value*
	No.	*%*	No.	%	
Total					
Age					0.08
≤40	12	24.5	7	10.9	
40–49	21	42.9	22	34.4	
50–59	8	16.3	16	250	
≥60	8	16.3	19	29.7	

Tumor size					0.7
T1	8	57.1	32	61.5	
T2-T3	6	42.9	20	38.5	

Grading					0.5
I	0		4	7.4	
II	7	50.0	26	48.1	
III	7	50.0	24	44.4	

Cerb2					0.3
Positive	1	6.7	9	15.8	
Negative	14	93.3	48	84.2	

Receptor status					0.2
Positive	9	64.3	45	80.4	
Negative	5	35.7	11	19.6	

**Table 4:** The DOBI-level.

Level	Score	*N *° Total	Cancer	No cancer	PPV**	NPV***
1	0*	0	0	0	0	100%
2	<20	10	0	10	0	100%
3	20–24	14	3	11	22%	78%
4	25–37	56	39	17	70%	30%
5	≥38	33	32	1	97%	3%

*Absence of API.

**Positive predictive value.

***Negative predictive value.
